# Morpho-Physiological and Molecular Evaluation of Drought and Recovery in *Impatiens walleriana* Grown Ex Vitro

**DOI:** 10.3390/plants9111559

**Published:** 2020-11-13

**Authors:** Marija Đurić, Angelina Subotić, Ljiljana Prokić, Milana Trifunović-Momčilov, Aleksandar Cingel, Milorad Vujičić, Snežana Milošević

**Affiliations:** 1 Department of Plant Physiology, Institute for Biological Research “Siniša Stanković”, National Institute of Republic of Serbia, University of Belgrade, Bulevar despota Stefana 142, 11060 Belgrade, Serbia; heroina@ibiss.bg.ac.rs (A.S.); milanag@ibiss.bg.ac.rs (M.T.-M.); cingel@ibiss.bg.ac.rs (A.C.); snezana@ibiss.bg.ac.rs (S.M.); 2 Department for Agrochemistry and Physiology of Plants, Faculty of Agriculture, University of Belgrade, Nemanjina 6, 11080 Belgrade, Serbia; ljprokic@agrif.bg.ac.rs; 3 Department of Plant Physiology, Faculty of Biology, University of Belgrade, Studentski trg 16, 11000 Belgrade, Serbia; milorad@bio.bg.ac.rs

**Keywords:** abiotic stress, *Impatiens walleriana*, abscisic acid, molecular analysis, antioxidants

## Abstract

This study was carried out to examine the drought effect on development, physiological, biochemical and molecular parameters in *Impatiens walleriana* grown ex vitro. Experiment design included three treatments: Control plants—grown under optimal watering (35%–37% of soil moisture content), drought-stressed plants—non-irrigated to reach 15% and 5% of soil moisture content and recovery plants—rehydrated for four days to reach optimal soil moisture content. Drought reduced fresh weight, total leaf area, as well as dry weight of *I. walleriana* shoots. Drought up-regulated expression of abscisic acid (ABA) biosynthesis genes *9-cis-epoxycarotenoid dioxygenase 4* (*NCED4*) and *abscisic aldehyde oxidase 2* (*AAO2*) and catabolic gene *ABA 8′-hydroxylase 3* (*ABA8ox3*) which was followed by increased ABA content in the leaves. Decrement in water potential of shoots during the drought was not accompanied with increased amino acid proline content. We detected an increase in chlorophyll, carotenoid, total polyphenols and flavonols content under drought conditions, as well as malondialdehyde, hydrogen peroxide and DPPH (1,1′-diphenyl-2-picrylhydrazyl) activity. Increased antioxidant enzyme activities (superoxide dismutase, peroxidase and catalase) throughout drought were also determined. Recovery treatment was significant for neutralizing drought effect on growth parameters, shoot water potential, proline content and genes expression.

## 1. Introduction

Drought is an abiotic stress factor that adversely affects plant growth and development, manifesting its effects from the cell to the whole organism level. Moreover, the drought is considered to be crucial in reducing plant productivity compared to all other environmental factors [1,2,3,4].

Reduced cell water potential and turgor, photosynthesis, transpiration, nutrient uptake and numerous metabolic processes are common consequences of drought effects on plant growth and development [1]. Decreased content of photosynthetic pigments during drought limit photosynthesis and certainly represent the interest in almost all research on this subject [5,6]. Drought-induced production of reactive oxygen species (ROS) plays an important role in oxidation of proteins, membrane lipids, DNA and RNA [2]. The imbalance between production and removal of ROS forms as a consequence of oxidative stress, is caused by a large number of primary stress factors, such as drought [2,7]. Reactive oxygen species include hydrogen peroxide (H_2_O_2_), superoxide anion radicals (O^2−^), hydroxyl radicals (OH•), singlet oxygen (^1^O_2_) and nitric oxide (NO) [2,7]. Of all stated, the hydroxyl radical is the most reactive and is produced in the Haber–Weiss reaction from O^2−^ and H_2_O_2_, and in the Fenton reaction of H_2_O_2_ in the presence of bivalent iron [7]. As there is no enzymatic reaction that can eliminate this radical, O^2−^ and H_2_O_2_ content in the cells must be strictly controlled by the activity of antioxidant system defense. One of the most studied ROS effects on the structure and function of cells is the peroxidation of membrane lipids. Malondialdehyde (MDA), one of the end products of lipid peroxidation, is considered as an indicator of oxidative stress in cells and its content reflects the degree of membrane lipid damage [7]. In addition to toxicity, ROS are important signaling molecules that participate in plant responses to abiotic stresses [8].

The sensitivity of plants to drought depends on stress degree, stress duration, effects of other stress factors, plant species and their developmental stages [4]. Plants have evolved three major drought resistance mechanisms: Drought escape, drought avoidance and drought tolerance [9,10]. Resistance mechanisms lead to adaptive changes in plant growth and physio-biochemical and molecular processes. Using these different strategies, plants try to cope with drought and survive unfavorable periods and often combine strategies to confer drought resistance. Many plants complete their life cycle before encountering local or seasonal drought, and in that way “escape” from stress. Drought avoidance means the ability of a plant to maintain high water potential in tissues even in the drought period. This is accomplished through rapid stomatal closure, reducing leaf area and enhancing the water uptake through well-developed root system [4,10,11]. In seed plants, particularly angiosperms, regulation of stomatal movement and transpiration is controlled by plant hormone abscisic acid (ABA) [12,13]. During drought, increase in ABA content could be the result of increased ABA biosynthesis and/or decreased ABA catabolism [14,15]. Biosynthesis of ABA is well established and branches from the terpenoid biosynthesis pathway in plastids [14]. The main regulatory step in ABA biosynthesis is encoded by *9-cis-epoxycarotenoid dioxygenase* (*NCED*) gene. The product of *NCED* gene expression is the enzyme 9-cis-epoxycarotenoid dioxygenase that cleaves the carotenoid precursors *cis*-violaxanthin and *cis*-neoxanthin to produce xanthoxin, first C15 precursor of ABA. Through oxidation, xanthoxin is then converted to ABA aldehyde in cytoplasm. Abscisic aldehyde oxidase, product of *abscisic aldehyde oxidase* (*AAO*) gene, catalyzes the final step in ABA biosynthesis converting ABA aldehyde in the biologically active form ABA. Likewise, during drought ABA could be released from sugar-conjugated forms such as ABA glucosyl estars by β-glucosidase activity. Conjugation is a way of inactivating ABA in plant cells [16]. Hydroxylation of ABA to phaseic acid is the main ABA catabolic pathway catalyzed by ABA 8′-hydroxylase enzymes which are encoded by cytochrome P450 monooxygenase gene family, *CYP707A* [14,15]. The processes of ABA biosynthesis, conjugation and catabolism act in a coordinated manner to control the ABA level in plants. During drought ABA induces stomatal closure, thereby reducing transpirational water loss. Increased ABA content during drought affects the stomatal closure by regulating the ionic transport in the guard cells through Ca^2+^ dependent or Ca^2+^ independent pathways [17]. In both cases, the plasma membrane of guard cells is depolarized through inhibition of H^+^-ATPase activity, inhibition of K^+^ influx channels, and activation of the anion channel [18,19]. Moreover, depolarization activates K^+^ efflux channels which in turn leads to a decrease in net ion concentration in guard cells. Fewer ions increase water potential in guard cells, which becomes higher than in surrounding cells. A gradient in water potential between the guard and surrounding cells enables water transport from guard to surrounding cells [19]. Losing water from guard cells decreases the cell turgor pressure which is followed by the closure of the stomata apparatus. The opposite effect of drought on plants stomatal behavior and ABA content has a rehydration process [20]. Drought tolerance implies the ability of a plant to maintain normal physiological functions even at the prolonged drought period and lower water potential in tissues. This is achieved mainly by the expression of specific genes that provide the accumulation of osmoprotectants and the components of the antioxidant system defense [1]. The accumulation of a large number of these components during drought is increased, including the amino acid proline [21], carotenoids [22], phenolic compounds [23,24] and antioxidant enzymes [25]. Antioxidant enzymes, such as superoxide dismutase (SOD), peroxidase (POX) and catalase (CAT) remove ROS from cells and reduce oxidation [7]. These enzymes are crucial to control O^2−^ and H_2_O_2_ level in cells, preventing forming of the most harmful hydroxyl radicals.

*Impatiens walleriana* (Balsaminaceae) is one of the three *Impatiens* species (beside *Impatiens hawkeri* and *Impatiens balsamina*) which are commercially produced in Serbia for many years. Due to its decorative traits and long flowering period, *I. walleriana* belongs to the most popular horticultural species all over the world [26]. Plants have high requirements for the presence of water in the substrate, which deficiency leads to a rapid drop in cell turgor pressure and tissue dehydration. This is the main problem in commercial production and market placement of these plants. Drought has detrimental effects on *I. walleriana* growth and development [27,28,29,30,31,32]. In this work, we have investigated the drought and recovery effect on growth, tissue water potential and proline and antioxidants content as well as activity of antioxidant enzymes in *I. walleriana* grown ex vitro. Similarly, we examined the effects of drought stress and recovery treatment on ABA content and expression of ABA metabolic genes. As plants responses to drought depend on many factors, in the present work we hypothesized differences in *I. walleriana* response to drought and recovery treatments on morpho-physiological, biochemical and molecular level.

## 2. Results

### 2.1. Soil Moisture Content Changes after the Onset of Drought Stress

**Figure 1:**
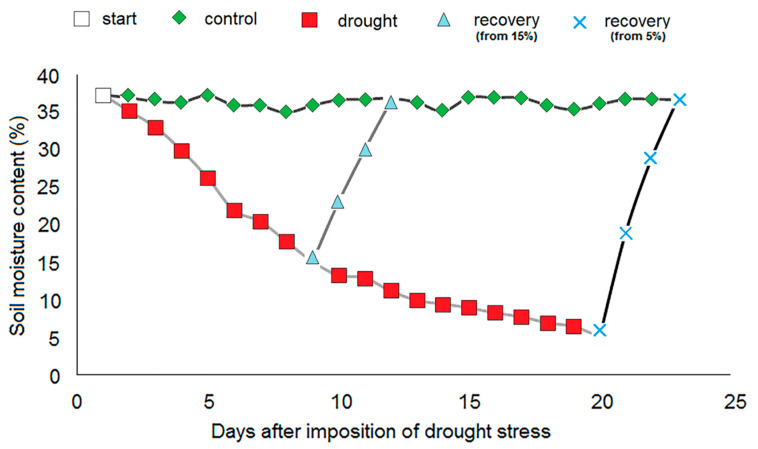
Changes in soil moisture content after imposition of drought stress for control, drought-stressed and recovery group of *I. walleriana*.

Changes in soil moisture content after the imposition of drought stress are presented in Figure 1. Drought stress was imposed on 44 days old *I. walleriana* and soil moisture content amounted 37% at this point. As shown in the Figure 1, control plants were grown on well-watered soil constantly during the experimental period while the two other plant groups were not irrigated to reach targeted soil moisture percentage (15% and 5%). From optimal soil moisture content in control plants (35%–37%) it was necessary for 9 days to achieve 15%, and 20 days to get 5% of soil moisture content. Recovery of drought-stressed plants was achieved with four days watering for two drought point (15% and 5%).

### 2.2. Changes in the Fresh Weight (FW), Total Leaf Area and Dry Weight (DW) in I. walleriana Subjected to Drought Ex Vitro

**Figure 2:**
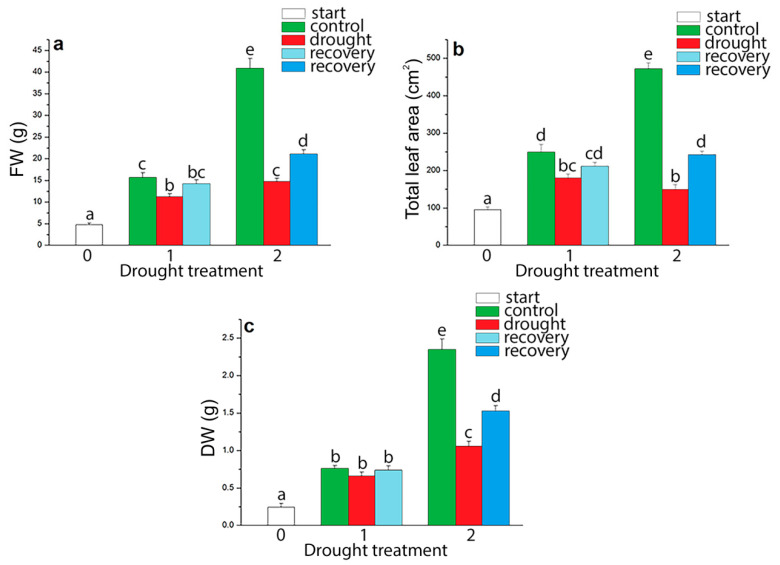
Growth parameters in drought-stressed *I. walleriana* grown ex vitro: Changes in shoots fresh weight (FW) (**a**), total leaf area (**b**) and shoots dry weight (DW) (**c**) were examined at the “start point”, 15% and 5% of soil moisture content—0, 1 and 2, respectively; results represent mean ± SE (*n* = 8). The letters indicate statistically significant differences based on the Fischer LSD test (*p* ≤ 0.05).

Reduced FW of shoots under drought was observed at both drought points, with the most expressed effects at 5% of soil moisture content (Figure 2a). At this point, FW was reduced by 63.91% in comparison to the control plants. Furthermore, at the lowest amount of water in a substrate, the effects of drought on total plant leaf area were the most pronounced. Leaf area of plants at 5% of soil moisture content was lesser by 3.17 times in comparison to the control plants (Figure 2b). Measurement of DW point to an expected decrease of DW in drought-stressed *I. walleriana* (Figure 2c) and DW was reduced for 54.96% in *I. walleriana* at 5% of soil moisture content. Recovery treatment had a positive impact on growth parameters in drought-stressed *I. walleriana*. FW, total leaf area and DW increased for 1.43, 1.63 and 1.44 times, respectively, in *I. walleriana* recovered from 5% of soil moisture content.

**Figure 3:**
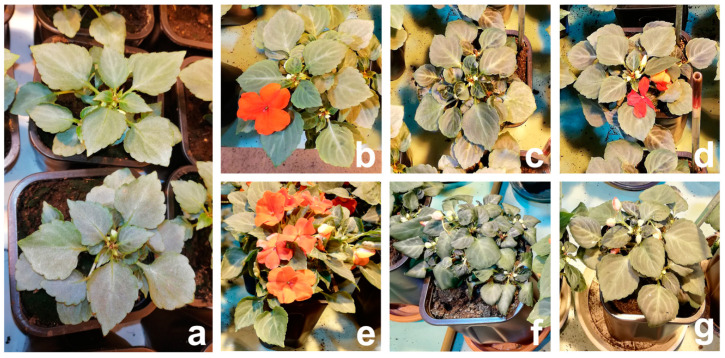
Morphological differences between *I. walleriana* at “start point“ of drought stress imposition (**a**); 15% of soil moisture content—control (**b**), drought-stressed (**c**) and recovered plants (**d**), and at 5% of soil moisture content—control (**e**), drought-stressed (**f**) and recovered plants (**g**).

Drying to 15% and 5% of soil moisture content induced morphological changes in *I. walleriana* (Figure 3) measured through above mentioned growth parameters (Figure 2). Control plants for two drought point (Figure 3b,e) had higher biomass comparing to stressed plants (15% and 5%) (Figure 3c,f) while the recovery plants of two drought point (Figure 3d,g) had a higher biomass than stressed plants. In comparison to control, height was reduced, and flowering was delayed in stressed plants (unmeasured, just noticed parameters). Further, at 5% of soil moisture, leaves of drought-stressed *I. walleriana* had changed morphology manifested through the wilting and rolling (Figure 3f).

### 2.3. ABA Content in Drought-Stressed I. walleriana

**Figure 4:**
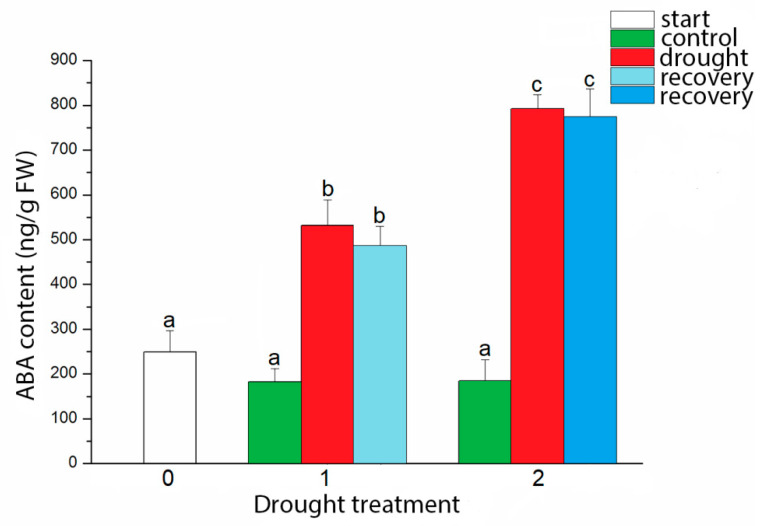
Effect of drought on abscisic acid (ABA) content in *I. walleriana* grown ex vitro. The “start point”, 15% and 5% of soil moisture content—0, 1 and 2, respectively; results represent mean ± SE (*n* = 8). The letters indicate statistically significant differences based on the Fischer LSD test (*p* ≤ 0.05).

With increasing drought intensity endogenous ABA content also increased (Figure 4). In drought-stressed plants ABA content was higher for 2.92 and 4.3 times (respectively for 15% and 5% of soil moisture content) than in control plants. Recovery treatment for four days did not induce a statistically significant reduction in ABA content, in comparison to the drought-stressed plants.

### 2.4. Expression of ABA Metabolic Genes in Drought-Stressed I. walleriana

**Figure 5:**
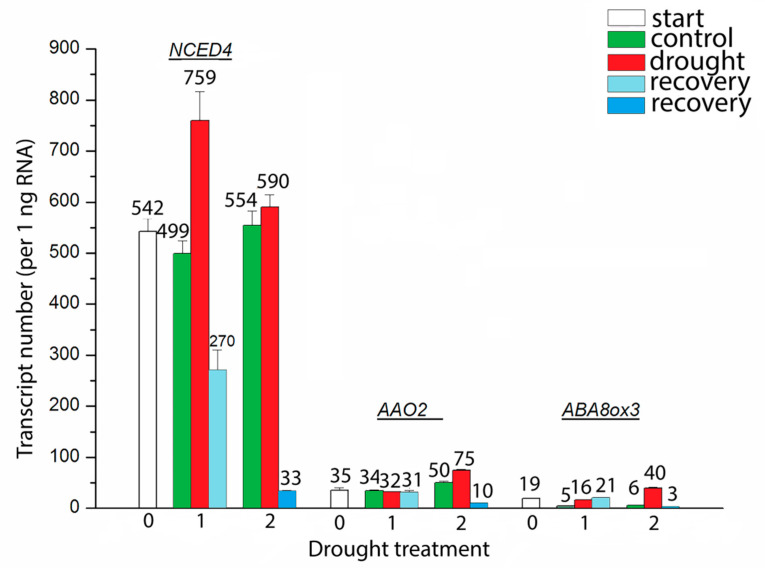
The effect of drought on *9-cis-epoxycarotenoid dioxygenase 4* (*NCED4*), *abscisic aldehyde oxidase 2* (*AAO2)* and *ABA 8′-hydroxylase 3* (*ABA8ox3*) gene expression in *I. walleriana* grown ex vitro. The “start point”, 15% and 5% of soil moisture content—0, 1 and 2, respectively; results represent mean ± SE (*n* = 8).

Expression analysis of ABA metabolic genes (*9-cis-epoxycarotenoid dioxygenase 4* (*NCED4*), *abscisic aldehyde oxidase 2* (*AAO2*) and *ABA 8′-hydroxylase 3* (*ABA8ox3*)) revealed amplification of specific fragments in *I. walleriana* leaves (Figure 5). At 15% of soil moisture content the number of *NCED4* transcript copies were drastically increased, while on 5% of soil moisture content transcript number decreased but still was higher than in control plants. On the other hand, *AAO2* gene expression was the most pronounced at 5% of soil moisture content. Number of copies of *NCED4* transcript per 1 ng RNA varied from 759 to 590 at 15% and 5% of soil moisture, respectively. For *AAO2* gene number of transcript copies varied from 32 to 75, respectively, for two drought points. Likewise, the number of copies for *ABA8ox3* in drought was lower in comparison with *NCED4* and *AAO2* expression. At 15% and 5% of soil moisture content number of copies of *ABA8ox3* varied from 16 to 40 per 1 ng of RNA. Recovery treatment decreased *NCED4* transcript number at both drought points in *I. walleriana*, while effects on *AAO2* transcript number reduction was most pronounced at 5% of soil moisture. Recovery was slightly induced and then decreased *ABA8ox3* transcript number at 15% and 5% of soil moisture, respectively.

### 2.5. Shoot Water Potential and Proline Content in Drought-Stressed I. walleriana Grown Ex Vitro

**Figure 6:**
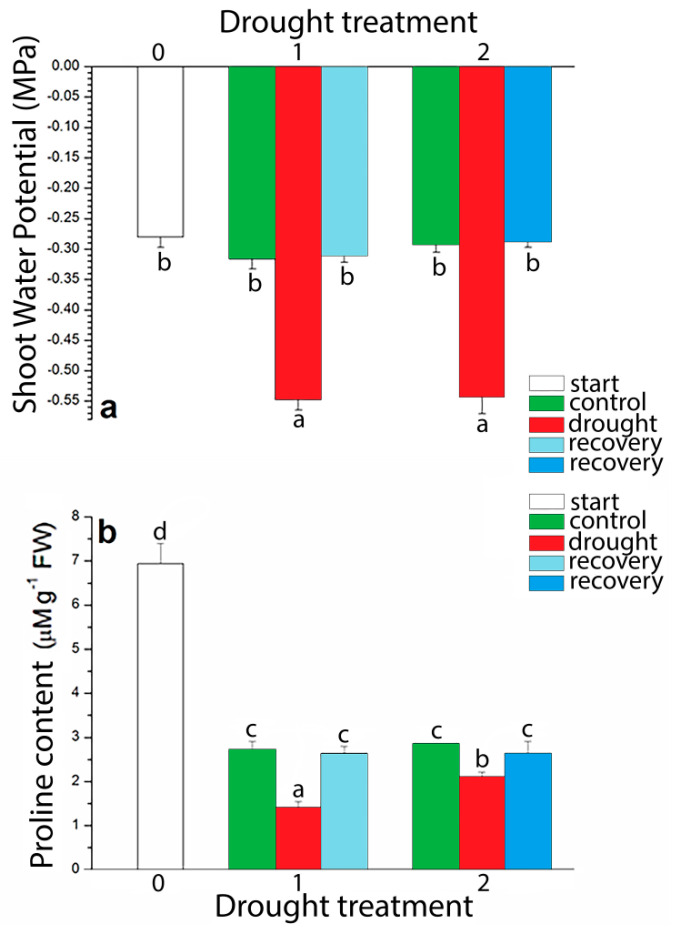
The effect of drought on shoot water potential (**a**) and proline content (**b**) in *I. walleriana* grown ex vitro. The “start point”, 15% and 5% of soil moisture content—0, 1 and 2, respectively; results represent mean ± SE (*n* = 8). The letters indicate statistically significant differences based on the Fischer LSD test (*p* ≤ 0.05).

Shoot water potential decreased in drought by 1.73 and 1.85 times below control values at two drought points in *I. walleriana*, respectively (Figure 6a). On the other hand, recovery treatment increased shoot water potential of drought-stressed plants to the control values. In order to determine the degree of osmotic adaptation of *I. walleriana* under drought conditions, the content of the amino acid proline in leaves was evaluated. Before beginning the drying period proline content was the highest (6.94 μM g^−1^ FW) and decreased with the duration of experimental period. Compared to the control plants, an unusual decrement in proline content in *I. walleriana* was observed at both induced drought points (Figure 6b). Proline content was lower by 48.22% and 26.46% in comparison to control plants at 15% and 5% of soil moisture content, respectively. Recovery of plants completely increased proline content up to the level of control values for both drought points.

### 2.6. Total Polyphenols, Flavonols and 1,1′-diphenyl-2-picrylhydrazyl (DPPH) Activity in Drought-Stressed I. walleriana

**Figure 7:**
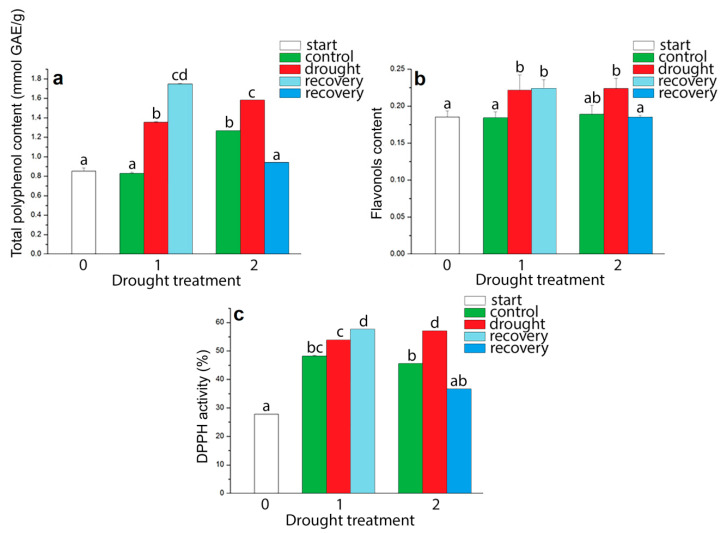
The effect of drought on total polyphenols (**a**) and flavonols (**b**) content, and 1,1′-diphenyl-2-picrylhydrazyl (DPPH) activity (**c**) in *I. walleriana* grown ex vitro. The “start point”, 15% and 5% of soil moisture content—0, 1 and 2, respectively; results represent mean ± SE (*n* = 8). The letters indicate statistically significant differences based on the Fischer LSD test (*p* ≤ 0.05).

Total polyphenols content in *I. walleriana* increased during drought (Figure 7a). In comparison with control plants total polyphenol content was higher by 64.02% and 24.87% at 15% and 5% of soil moisture, respectively. In *I. walleriana* recovered from first drought point total polyphenols content increased by 24.99% and decreased by 59.59% in plants recovered from second drought point, compared to drought-stressed plants. Recovered plants from second drought point had a lower total polyphenol content than control plants group. As shown on Figure 7b, drought stress also induced increment in flavonols content in leaves of *I. walleriana* grown ex vitro. Flavonols content was higher by 20.41% and 18.54% at 15% and 5% of soil moisture content, respectively, in comparison to the control plants. The rehydration process decreased flavonols content to slightly below the control values after the most pronounced drought stress. Drought stress also induced increment in antioxidant activity measured by DPPH method. Antioxidant activity was higher with 5.60% and 11.50% at 15% and 5% of soil moisture compared to control plants, while in recovered plants noticed a similar trend as for total polyphenol and flavonols content (Figure 7c). Plants recovered from first drought point increased antioxidant activity for 3.84% in comparison to drought-stressed plants, while recovery from second drought point decreased antioxidant activity for 20.35% (lower than in control plants for 8.86%).

### 2.7. Spectrophotometrical Quantification of Photosynthetic Pigments, Hydrogen Peroxide and Malondialdehyde in Drought-Stressed I. walleriana Grown Ex Vitro

**Figure 8:**
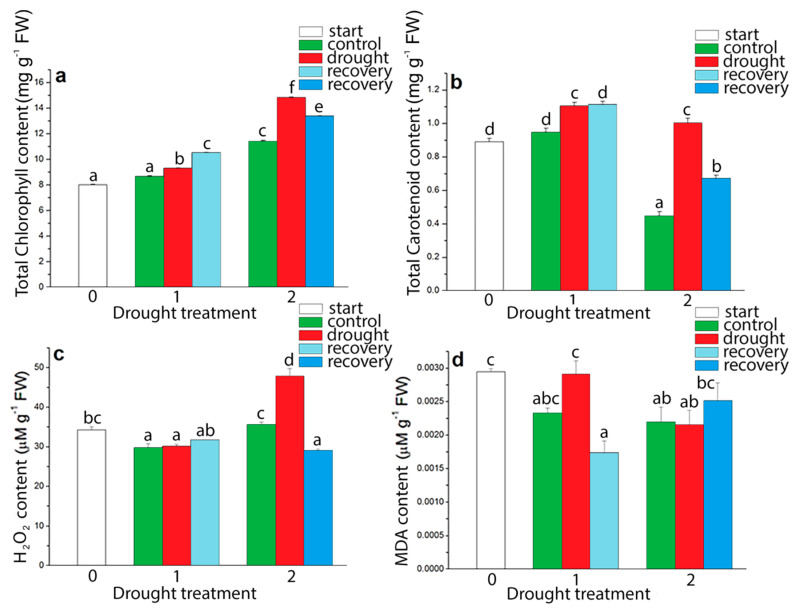
The effect of drought on total chlorophyll (**a**), total carotenoid (**b**), H_2_O_2_ (**c**) and malondialdehyde (MDA) (**d**) content in *I. walleriana* grown ex vitro. The “start point”, 15% and 5% of soil moisture content—0, 1 and 2, respectively; results represent mean ± SE (*n* = 8). The letters indicate statistically significant differences based on the Fischer LSD test (*p* ≤ 0.05).

Spectrophotometrical analysis showed that drought induced changes in total chlorophyll and carotenoids content in *I. walleriana* (Figure 8a,b). Drought-induced changes in both, chlorophyll and carotenoid content, had a similar trend. The increment in the content of the total amount of chlorophyll and carotenoid in *I. walleriana* was observed at both drought points. Compared to the control plants, chlorophyll increment was 29.85% higher at 5% of soil moisture content (Figure 8a), while the carotenoid increment at the same soil moisture content was more pronounced. Carotenoid content was 2.23 times higher in *I. walleriana* leaves at 5% of soil moisture content than in control plants (Figure 8b). The recovery of plants had a similar effect on the chlorophyll and carotenoid content in the leaves of drought-stressed *I. walleriana*. Recovery from the first drought point (15%) slightly increased chlorophyll and carotenoid content, while the recovery from the second drought point (5%) decreased chlorophyll and carotenoid content in *I. walleriana*.

As the production of ROS increased during drought, the content of H_2_O_2_ was also determined (Figure 8c). A statistically significant increment in H_2_O_2_ content in *I. walleriana* was observed by exposing the plants to the progressive drought (Figure 8c). Compared to control plants, the H_2_O_2_ content was increased by 34.41% at 5% of soil moisture while recovery treatment reduced H_2_O_2_ content below the control level at this point. The effect of different drought intensities on the degree of lipid peroxidation in *I. walleriana* was determined based on the MDA content (Figure 8d). As in the case of proline content (Figure 6b), very high MDA content in *I. walleriana* leaves was observed at the start point of imposition of drought stress. A significant increment in MDA content was observed when the soil moisture in the substrate reached 15%. The increment in MDA content was 25.13% higher in comparison to control plants. With increasing drought intensity, the MDA content dropped to a level similar to that of control plants. From the first drought point (15%), recovery managed to lower the MDA content in comparison to control plants, but from the second drought point (5%), recovery induced a slight jump (for 14.69%) in MDA compared to control group of plants.

### 2.8. Superoxide Dismutase, Catalase and Peroxidase Activity in Drought-Stressed I. walleriana

**Figure 9:**
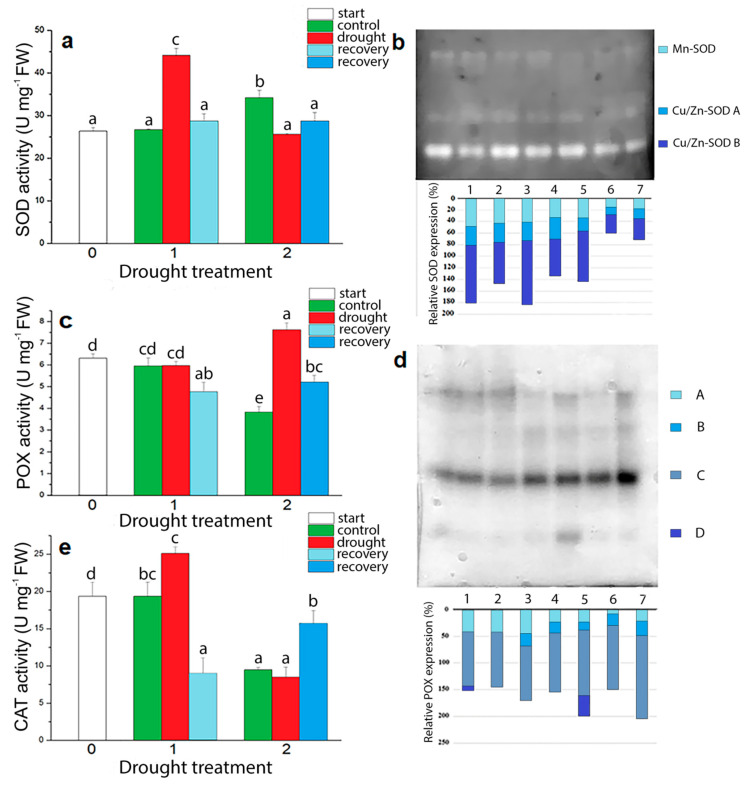
(**a**) superoxide dismutase (SOD) activity; (**b**) SOD isoforms and their relative expression (%); (**c**) peroxidase (POX) activity; and (**d**) POX isoforms and their relative expression (%) and catalase (CAT) activity (**e**) in drought-stressed *I. walleriana* grown ex vitro. (**a**,**c**,**e**)—the “start point”, 15% and 5% of soil moisture content—0, 1 and 2, respectively; (**d**,**e**)—the “start point” (1), 15% of soil moisture content—control (2), drought (3), recovery (4) and 5% of soil moisture content—control (5), drought (6) and recovery (7). Results represent mean ± SE (*n* = 8). The letters indicate statistically significant differences based on the Fischer LSD test (*p* ≤ 0.05).

The effects of drought on total SOD activity of *I. walleriana* are shown on Figure 9a, while on Figure 9b are presented SOD isoforms and their relative expression. At 15% moisture content in the substrate, plants drastically increased SOD activity, which was 1.65-fold higher than control values. With drought stress progression, SOD activity decreased in comparison to control plants. Recovery of plants contributed to the reduction of SOD activity to the level in control plants at 15% of soil moisture content. *I. walleriana* expressed one Mn-SOD and two Cu/Zn-SOD (A and B) isoforms in all treatments with the strongest activity of Cu/Zn-SOD B isoform.

At the drought stress start point, higher POX (Figure 9c) and CAT (Figure 9e) activities were observed in comparison to both controls. These results correspond with proline (Figure 6b) and MDA content changes (Figure 8d) at the same point. There were no significant changes in POX activity between control and drought-stressed *I. walleriana* plants at 15% of soil moisture content, while the CAT activity at the same point was higher for 29.77% in drought-stressed plants. The opposite trend was observed in plants at 5% of soil moisture content. In drought-stressed plants POX activity was almost two time higher while the CAT activity was not significantly changed in comparison to control plants. Recovery from the first drought point decreased POX and CAT activities, while recovery from the second drought point decreased POX activity and, at the same time, increased CAT activity. Different POX isoforms in *I. walleriana* subjected to drought are presented in Figure 9d. Total four POX isoforms were detected on gel and, among them, D isoforms are specific for non-stressed *I. walleriana* plants.

## 3. Discussion

The drought is considered as the most represented problem in horticulture, agriculture and forestry. Initially, plants response to drought in a similar way, and that is manifested through inhibition of various parameters of growth and development [10,33]. In this work, we defined the effects of two drought points and recovery on the morphological, physiological, biochemical and molecular parameters of the *I. walleriana* grown under controlled ex vitro conditions. 

It is widely known that drought reduces plant growth primarily due to the inhibition of cell division and enlargement, which is considered as one of the most sensitive processes during drought. With increasing drought intensities, FW and DW of *I. walleriana* were reduced. Similar results for *I. walleriana* were described by authors [28,29,32], as well as for *I. balsamina* [34] and petunia [35]. Drought significantly reduced the total leaf area of *I. walleriana* cultivated ex vitro, as previously was recorded in petunia and cut rose [35,36]. Leaf area reduction is a strategy that prevents excessive water loss from tissues during drought stress. Reduced leaf area mostly means less water wasted by transpiration through stomata. Similarly, to avoid or mitigate the drought effects, many plants completely reject leaves or reduce the transpiration surface in a different way, e.g., by curling leaves [11]. In this experimental work was noticed that flowering was delayed in drought-stressed *I. walleriana* which reduced reproductive capacity. Authors [27] and [28] previously described that drought-stressed *I. walleriana* grown in the greenhouse conditions reduced number of flowers.

With increment in drought intensities, ABA content also increased in *I. walleriana* leaves. ABA increment during drought is generally expected, considering ABA’s roles in the stomatal conductance and transpiration rate control during drought [37,38]. Increment in ABA content in *I. walleriana* leaves was correlated with gene expression analysis of ABA biosynthesis and catabolic genes. Up-regulated *NCED4* and *AAO2* genes in drought-stressed *I. walleriana* were observed, as well as *ABA8ox3* gene expression. Up-regulated catabolic gene *ABA8ox3* in drought-stressed *I. walleriana* may be resulted from the feed-back regulation by the increasing of ABA content and *NCED4* and *AAO2* expression. The similar results were previously described for many plants. Increased expression of ABA biosynthesis genes *NCED* and *TAO1* (*AAO* gene family) were observed in drought-stressed tomato [39] as well as *NCED3* in drought-stressed rice [40]. Authors [40] also analyzed expression of three *ABA8ox* genes in drought-stressed rice and showed increased expression in drought for *ABA8ox3.* Rehydration decreased expression for *NCED3* but *ABA8ox3* was instantly induced in rice. This relationship in expression of ABA biosynthesis and catabolism in the rehydration process allowed decreased ABA content in rice leaves. In study with *Pisum sativum* [41] it was observed that rapidly imposed drought stress did not induce expression of *PsAAO3* gene, while its expression is stimulated under progressively imposed drought by withholding watering. On the other hand, high level of *NCED2* and *NCED3* genes transcripts was observed in both drought treatments with differences in organ-specific expression. Recent study of drought effect on ABA content and ABA metabolic genes expression in *Pinus sylvestris* and *Picea abies* showed that ABA content during drought was not regulated on transcriptional, but more likely on posttranscriptional level [42]. Authors concluded that ABA level in roots and needles were not determined by the levels of ABA biosynthetic genes expression. Recovery treatment for four days did not restore ABA content in drought-stressed *I. walleriana*, but we suppose that ABA content in recovering plants would certainly drop to the control level during the time. Similar rehydration results in relationship to ABA content are described previously in the early nineties [43] and later for grapevines [44], and characterized as ABA “after effect” on stomata and transpiration rate. This means that ABA synthesized during drought could control stomatal conductance and transpiration rate in early recovery phases until the moment when its content starts to decrease. When ABA content starts to decrease in the rehydration process, stomatal conductance and transpiration rate starts to increase. Relationship between expression of ABA biosynthesis and catabolism genes in recovery treatment could be the reason for unchanged ABA content in leaves of drought-stressed and recovered *I. walleriana.* It could be expected that *ABA8ox3* gene expression increased after certain period of watering to reduce ABA content, but as well, main catabolism role could have another gene from the *CYP707A* family in *I. walleriana*. Moreover, ABA may be inactivated by sugar-conjugation and forming ABA glucosyl esters.

In addition to the role in transpiration rate control, ABA’s role in drought tolerance could express through up-regulation of different genes involved in plants adaptive response to drought such as genes for water transporters—aquaporins (AQP), phenolic and antioxidant compounds [45,46,47].

Decreased shoot water potential was observed in *I. walleriana* exposed to drought. The reduction of water potential during drought was also recorded in cotton [48] and *Chromolaena odorata* leaves [49]. For the water trending in the direction of the substrate → root → stem → leaf → atmosphere, it is necessary to have a difference in water potential between these parts. Drought can reduce the water potential in the substrate below the water potential value in the root xylem, thereby preventing its adoption. During the evolution, plants have evolved mechanisms of tolerance for various types of stress, which include, among others, mechanisms that provide water retention in tissues during dehydration [50]. To allow the absorption and movement of water, many plant species accumulate specific metabolites that further lower the water potential in cells that can reach values below the water potential of the substrate [51]. In this way, a gradient is created in the water potential between the substrate and the root, which ensures the water flow through the plant. Such substances are generally accepted as compatible solutes or osmoprotectants. The osmoprotectants include amino acids, sugars, sugar alcohols (xylitol, sorbitol and mannitol), polyamines and quaternary ammonium compounds [50,51]. Osmoprotectants also protect cellular structures, enzymes and scavenge ROS [51]. Amino acid proline is often considered as a stress indicator because in most plant species increased proline accumulation is associated with plant responses to different types of abiotic stress [51]. Increased proline content during drought was observed in many plant species [52,53] but our results demonstrated that proline has no significant osmoprotectant role in drought-stressed *I. walleriana*. Drought stress could negatively regulate the proline biosynthesis affecting the activity of enzymes involved in the biosynthesis of this amino acid. Nevertheless, the absence of a positive correlation between proline content and drought stress in *I. walleriana* may reflect the predominance of some other osmotic adjustment mechanism.

Polyphenols are the largest group of plant secondary metabolites with different roles in plants growth and development, including plants responses to biotic and abiotic stress factors [24]. Members of polyphenols are involved in seed germination, cell division, nutrient uptake, pollen development, signal transduction and plant interaction with environment. All polyphenols originated from phenylalanine and, on basis of the presence of multiple phenolic structural units, are divided into several groups (phenolic acid, flavonoids, stilbenes and lignans). As antioxidants, polyphenols are very important in scavenging ROS forms in stressful conditions and preventing molecules oxidation and membranes peroxidation [24]. Increased accumulation of polyphenols in responses to drought was observed for many plants. Recent researches showed that drought induced increase in polyphenols and flavonoids in *Triticum aestivum* [54], three *Achillea species* [55], *Tymus vulgaris* [56], *Achilea pachycephala* [57] and *Hordeum vulgare* [58]. Increment in total polyphenols and flavonols (subgroup of flavonoids) in *I. walleriana*, indicated that drought positively regulates biosynthesis of phenolic compounds which plays important role in elimination of ROS and plant defense against drought. It is widely known that drought regulates many genes of phenylpropanoid metabolism to increase accumulation of phenolic compounds in plants [24]. Increased accumulation of total polyphenols and flavonols was accompanied with enhanced resistance of *I. walleriana* to oxidative stress. Similar pattern of accumulation of these secondary metabolites in recovered *I. walleriana* is dependent of previously imposed drought stress intensity.

Antioxidative activity of secondary metabolites in drought-stressed *I. walleriana* was showed through DPPH radical scavenging method. DPPH method is characterized as a rapid, simple and economic method, and is often used for evaluating antioxidative potential of antioxidants in different biological sources [59]. Antioxidants react with free radical to prevent oxidative process in its initiation, propagation or termination. Increased antioxidant activity based on DPPH assay was noticed in drought-stressed *I. walleriana* as well as in Serbian melliferous species [60], *Saccharum officinarum* [61], *Achillea species* [57], *Silybum marinum* [62] and many others.

Chlorophyll increment during drought is unusual because the lack of water in the tissues generally disturb chlorophyll biosynthesis. However, authors [63] have described chlorophyll increment in osmotic stress induced by polyethylene glycol in grass *Bouteloua gracilis* while in some ornamental plants, drought stress did not affect chlorophyll content [64]. We also observed an increase of chlorophyll content in drought-stressed *I. walleriana*. Theoretically, increased chlorophyll content may enhance the rate of photosynthesis. Obviously, that is not enough to overcome the problem of drought in the context of plant productivity, but resources can be used in the biosynthesis of various components in chloroplasts, such as osmoprotectants, fatty acids, starch or other compounds with a protective role in stress. Likewise, first reactions in ABA biosynthesis take place in chloroplasts, so maybe these protective mechanisms allow an increment in chlorophyll in drought. Similar explanation for unusual chlorophyll increment in *Bouteloua gracilis* under osmotic stress was provided by [63]. Since carotenoids have a photoprotective or an antioxidant role in plants, increased carotenoid content during drought is not unexpected. As in this experiment, increased carotenoid content during drought was also previously observed in *Helianthus annuus* [65] and *Vataire macrocarpa* [22].

Increased H_2_O_2_ content in stress conditions is considered as an indicator of the degree of oxidative stress in plants. In *I. walleriana* plants exposed to progressive drought, a drastic increment of H_2_O_2_ was observed. The similar results have also been described in *Achilea* sp. [55] and *Amaranthus tricolor* [66] exposed to drought stress. H_2_O_2_ is a non-radical ROS form that is synthesized in all cell compartments, including chloroplasts, peroxisomes, mitochondria, plasma membranes and apoplast. Plant cells, unlike animals, are tolerant to high concentrations of H_2_O_2_ and H_2_O_2_ treatments often increase the resistance of plants to different types of abiotic stresses [67,68]. The toxicity of H_2_O_2_ in the plant cells is reflected in its ability to form OH• [3]. In some plant species, such as the epiphyte *Guzmania monostachia*, there was observed a decline in the H_2_O_2_ content during drought [69]. The reason for this is the presence of crassulacean acid metabolism (CAM) metabolism at *G. monostachia*, which allows the accumulation of malate and reduction of photorespiration as a source of H_2_O_2_. In addition, very high glutathione reductase activity was observed in water deficit conditions in these epiphytes. Through lipid peroxidation, ROS disrupt the integrity and functionality of biological membranes. Lipid peroxidation products include various intermediates and final products like MDA, which is the most often mentioned and tested parameter in different stress conditions. Increased MDA content was observed in leaves of *I. walleriana* plants exposed to drought. The similar results have been described previously for *Vigna unguiculata* [70] and *Eucalyptus globulus* [71]. However, there are literature data describing MDA content has not changed in drought or even declined [55,72]. In this experiment, MDA content in the cells was not changed after the exposure of *I. walleriana* to progressive drought. Most probably, the reason for this is increased activity of antioxidative defense system whose components, both enzymatic and non-enzymatic, remove ROS forms and their products, thereby preventing cell death [73,74]. In progressive drought *I. walleriana* increased total polyphenol content, flavonols, DPPH activity, carotenoids, as well as POX activity. Increased content of antioxidants contributed to protection of cell membranes stability through preventing lipids disruption, which resulted in unchanged MDA content. A reason for a slight jump in MDA content after recovery from progressive drought, in comparison to MDA control values, could be attributed to *I. walleriana* metabolism plasticity to cope with drought stress. The components of the antioxidative defense system of plants, depending on the genotype and the causes of their induction, act in a way that at the given moment provides maximum protection [7]. Their activity may be increased, but also reduced or unchanged in stressful conditions [75]. If some component of the antioxidant protection system reduces its activity, the activity of another component increases, compensating for this initial lack of activity. In some plants in stressful conditions, the activity of non-enzymatic antioxidant factors is dominant, while in others it is attributed to enzymatic components. In any case, their functioning is complex and dynamic and depends on a large number of factors at a time, with the unchanged purpose of action.

Increased oxidative stress, altered the activity of antioxidant enzymes in *I. walleriana*. In most cases, elevated ROS concentration in the cells increased the activity of the enzymes that remove ROS in order to establish homeostasis in the organism. Superoxide dismutase converts the superoxide anion radical into H_2_O_2_, and present the first line of defense against ROS. Further, enzymes CAT and POX translate H_2_O_2_ to water and oxygen. Increased SOD activity was observed in *I. walleriana* as well as in *Glycyrrhiza glabra* [76], *Cerasus humilis* [77] and rice [78] as a response to oxidative stress. The variability in SOD activity caused by tissue dehydration, besides its increasing, also includes declining or unchanged activity [79,80]. Increased SOD in *I. walleriana* was followed by increased CAT activity in order to convert produced H_2_O_2_ to water and oxygen. That is actually the reason why determined H_2_O_2_ content at 15% of soil moisture was not significantly different between control and drought-stressed plants. Additionally, different SOD isoforms are expressed and detected in drought-stressed *I. walleriana*. SOD enzymes isoforms are classified by metal as a cofactor into three groups: Cu/Zn-SOD, Mn-SOD and Fe-SOD. In wheat, the Mn-SOD isoform plays a major role in the scavenging of superoxide radicals [81], while in sugarcane Fe-SOD were strongly induced in chloroplast during drought [82]. In this experiment three detected SOD isoforms (one Mn-SOD and two Cu/Zn-SOD) were expressed in all treatments. Differently expressed SOD isoforms during viral infection and virus elimination in *I. walleriana* were previously described by [26]. Catalase is considered as an energy-efficient, highly effective enzyme, not only because of its catalytic properties, but also because it is the only antioxidant enzyme that does not produce a new ROS form. Depending on the H_2_O_2_ content, CAT shows bifunctionality. When H_2_O_2_ content is lower than 1 μM, CAT has a peroxidative activity and demand for the electron donor for the reduction of H_2_O_2_. On the other hand, higher H_2_O_2_ content enabled a rapid degradation of H_2_O_2_ to water and oxygen [83]. In drought-stressed *I. walleriana* grown at 5% of soil moisture content, decreased SOD activity was followed by unchanged CAT activity in comparison to control plants. However, at this drought point high POX activity was observed and obviously POX has a main role in removing of high H_2_O_2_ content. Similarly, drought stress differently affects POX isoforms in *I. walleriana*, as described previously for *I. walleriana* grown in vitro under water deficit condition [32] and *Trifolium* sp. in a growth chamber [84]. Peroxidases have a higher affinity for H_2_O_2_ by eliminating it from different cell compartments using different electron donors. Ascorbate and glutathione peroxidase are the most involved in ROS detoxification, catalyzing the H_2_O_2_ reduction by electron donors, ascorbate and glutathione [85]. High POX activity, together with high polyphenols, flavonols and carotenoid content is a dominant defense component of antioxidant system in *I. walleriana* in progressive drought. In general, both enzymatic (SOD, POX, CAT) and non-enzymatic components (phenolic compounds, carotenoids) of antioxidant system defense are very important in *I. walleriana* adaptive responses to drought.

## 4. Materials and Methods

### 4.1. Plant Material and Experiment Design

The experiment was conducted from May to July 2018 in a growth chamber at Faculty of Agriculture, University of Belgrade. Seeds of *I. walleriana* (Xtreme Scarlet, Syngénta) were germinated on plates containing Klasman Potgrond H commercial substrate. The average temperature ranged from 22–25 °C (day) to 17 °C (night), with photoperiod of 16/8 h (day/night), 100% relative humidity and light intensity of 250 mmol m^−1^s^−1^. After seed germination, plants continued to grow under the same temperature, light intensity and photoperiod, but relative humidity was 55%–60%. One-month-old seedlings were transplanted into 10 by 10 by 13 cm plastic pots containing 450 g of the Klasman substrate and irrigated daily to reach an optimal soil moisture of 35%–37%. The drought stress was started after 14 days of transplanting *I. walleriana* growth in plastic pots under optimal watering. Control plants grow under optimal irrigation (35%–37% of soil moisture content) while two other plant groups were not irrigated to reach of 15% and 5% moisture in the substrate. There were recovery plant groups for both drought treatments, where the effects of drought on plants had been gradually neutralized. Recovery of stressed plants was achieved by watering for four days to optimal soil moisture content. The leaves were sampled from plants at “start point” (on time of beginning the drying period when soil moisture was 35%–37%), control, drought-stressed and recovered plants (seven treatment groups of *I. walleriana*). All samples were frozen in liquid nitrogen and then stored at −80 °C for further analyses.

### 4.2. Measurements of Soil Moisture Content, Flavonols, Shoots Water Potential, Fresh Weight of Shoots, Leaves Area and Dry Weight of Shoots

The soil moisture content was measured every morning (at 9 a.m.) with the Theta Probe (Type ML2x, Delta-T Devices Ltd., Cambridge, UK). The flavonols content in the leaves was measured by Dualex Scientific (FORCE-A, Orsay, France)—the optical sensor which provides a simple, fast and non-destructive measurement in plant leaves. Measurement of shoots water potential was carried out in the pressurized chamber under the pressure of nitrogen gas (Soil Moisture Equipment Corp., Santa Barbara, CA, USA). A pressure which leads to the first drops of xylem sap was used as the value of the water potential and was expressed in MPa. Subsequently, FW of shoots and total leaf area by area meter were measured (LI-3100 AREA METER, LI.COR. Lincoln, NE, USA). Shoots DW was measured after the samples drying for a few days on room temperature and then 48h at a temperature of 70 °C.

### 4.3. Determination of Abscisic Acid Content

ABA extraction from leaves was carried out as described by authors [39]. Measurement of ABA content was determined by ELISA method [86] using a specific MAC 252 monoclonal antibody for ABA [87]. Plate contents (Nunc: F96 Maxisorp immuno plate) were read at 405 nm by ELISA reader (Sunrise, Tecan. Männedorf, Switzerland).

### 4.4. Analysis of ABA Metabolic Genes Expression

#### 4.4.1. RNA Isolation and Real Time PCR (RT-PCR)

Total RNA was isolated from *I. walleriana* leaves (100 mg), according to the [88]. Total RNA was quantified with a NanoDrop spectrophotometer (NanoPhotometer^®^ N60, IMPLEN, Munich, Germany) and its quality and integrity were estimated by electrophoretic separation on 1.5% agarose gel. To eliminate traces of DNA, RNA was treated with DNase I (Thermo Fisher Scientific, Waltham, MA, USA) at 37 °C for 10 min, according to the manufacturer’s protocol. cDNAs were synthesized in reverse transcription reaction (RT) from 1 µg of total RNA. Reaction mixture for RT in volume of 21 µL, contained 10 µL of total RNA (0.1µg/µL), 25 mM MgCl_2_, 1 mM dNTP, inhibitor RNA-asa (20 U/µL), random hexamers (50 µM) and 15 U of MultiScribe^®^ transcriptase.

#### 4.4.2. Quantitative Real Time PCR (qRT-PCR)

Abscisic acid biosynthesis (*NCED4* and *AAO2*) and catabolic (*ABA8ox3*) genes expression were measured by quantitative real time RT-PCR using SYBR green in QuantStudio 3 Real-Time PCR System (Applied Biosystems). Reaction mixture of 10 µL containing 1 μL of RT reaction product, appropriate forward and reverse primers and Maxima SYBR Green/Rox qPCR Master Mix (Thermo Fisher Scientific, Waltham, MA, USA). Thermal cycling conditions for qRT-PCR include: Initial denaturation on 95 °C, then 40 cycles of denaturation (95 °C for 30 s), annealing (60 °C for 30 s) and extension (72 °C for 30 s). Annealing temperature of 60 °C was the same for all used primers. For each sample, qRT-PCR was performed in triplicate. Sequences of appropriate transcripts were obtained from the sequenced leaf transcriptome of *I. walleriana* (www.genomix4life.com) and identified using Trinotate (combines homology search to known sequence data (BLAST/SwissProt), protein domain identification (HMMER/PFAM), protein signal peptide and transmembrane domain prediction (signalP/tmHMM) and leveraging various annotation databases (eggNOG/GO/Kegg databases). Primer-BLAST (www.ncbi.nlm.nih.gov/tools/primer-blast) was used for primer design while primer properties were checked by NetPrimer software. Primer specificity was confirmed by electrophoretic analysis of RT-PCR products, and melting curve analysis. Gene with constitutive expression—actin (*ACT1*), was used as an endogenous control. The forward primer 5’-CCGACGTGCCTATCTTCTCC-3´ and reverse primer 5´-CACCTCATCTCCGCCTCATC-3´ were used for *NCED4* amplification (GenBank™ Accession No. MW087125); forward primer 5´-GTCTTCGCTCCAACATTCGC-3´ and reverse primer 5´- CCCCAACAGACTGCCTTCAT-3´ were used for *AAO2* amplification (GenBank™ Accession No. MW087126) while forward primer 5´-CTACATCAGCCACAGCCTCC-3´ and reverse primer 5´-CTCAGGACACAACTGCCACT-3´ were used for *ABA8ox3* amplification (GenBank™ Accession No. MW087127). Amplification of *ACT1* (GenBank™ Accession No. MW087128) was carried out with the forward primer 5´-GTGGTGGTGAAGGAGTAGCC -3´ and reverse primer 5´-TTCAGGTGATGGTGTGAGCC-3´. Absolute quantification was done using the standards for *NCED4*, *AAO2*, *ABA8ox3* and *ACT1* genes obtained by GeneJET Gel Extraction Kit (Thermo Fisher Scientific, Waltham, MA, USA ) according the manufacturer’s protocol. Standards were prepared as a serial dilution in a range from 10^9^ to 10^2^.

### 4.5. Spectrophotometric Analysis of Total Polyphenol Content (Folin–Ciocalteu Test) and Antioxidant Activity in Plants (DPPH Method)

Total polyphenol content was determined using Folin–Ciocalteu test (FC test) based on previously described method [89]. Polyphenols in plant extracts react with Folin–Ciocalteu reagents, forming blue colored complex that can be spectrophotometrically quantified. For this purpose, 200 mg of plant leaves was homogenized in liquid nitrogen with 1 mL of 96% ethanol. Extract was incubated for 60 min at room temperature and then centrifuged at 12,000× *g* for 15 min. Supernatant was transferred in new Eppendorf tubes. FC reagents solution was prepared by adding distillated water in FC reagents in a volume ration 2:1. Reaction mixture contained 300 µL of FC reagents solution, 1340 µL of deionized H_2_O and 60 µL of supernatant. The mixture was quick vortexed and left at room temperature for 5 min. Then into mixture was added, 300 µL of 20% Na_2_CO_3_ and left at room temperature for 90 min in dark conditions. Absorbance was measured at 765 nm. Gallic acid was used as a standard phenol. The mean of three readings was calculated and the total polyphenol content was expressed as mmol of gallic acid equivalents/g extract.

Antioxidant activity based on the concentration of DPPH radicals in plants was determined in leaf samples of *I. walleriana* prepared on the same way as for FC test. DPPH radical is a stable radical with maximum absorption at about 520 nm. In reaction with antioxidants stable radical is converted in non-radical form through reduction by hydrogen ion. DPPH reagent solution was prepared with methanol (4.5 mg of DPPH with 45 mL methanol). Absorbance of the prepared DPPH reagent solution should be less than 1.0. In supernatant volume of 100 µL was added 500 µL of methanol and 400 µL of DPPH reagent solution and reaction mixture was incubated at room temperature for 60 min in dark conditions. Degree of reduction of DPPH radicals was estimated through absorbance measurement at 520 nm. The scavenging capacity of the DPPH radical was calculated using the following equation: (%) = [1 − (A1 − A0)] × 100 where A1 is the absorbance of the sample and Ao is the absorbance of the blanc reaction (methanol instead of sample).

### 4.6. Spectrophotometric Analysis of Proline Content, Photosynthetic Pigments, Malondialdehyde and Hydrogen Peroxide

Quantification of proline, total chlorophyll, carotenoids, as well as MDA and H_2_O_2_ content were determined spectrophotometrically according to [27].

### 4.7. Protein Extraction and Enzyme Assays

Total soluble protein extraction, determination of protein concentration, as well as total POX and CAT activities were done according to [21] while SOD activity was determined as described by [27]. All enzymes activities were expressed as μmol min^−1^ mg^−1^ of soluble protein (U mg^−1^). Separating of SOD and POX isoforms were done by electrophoresis on polyacrylamide gel as described by [21].

### 4.8. Statistical Analysis

StatGraphics version 4.2 was used for statistical data processing (STSC Inc. Rockville, MD, USA). Statistical differences between treatments were assessed by one-way ANOVA while the mean differences were compared using the Fischer LSD test, with a statistical significance of *p* ≤ 0.05. Gels were analyzed by ImageJ program package.

## 5. Conclusions

Drought affects *I. walleriana* growth and development by modulating various physiological biochemical and molecular responses. Through inhibition of growth parameters, drought induced a decline in plant productivity with obvious morphological changes. Drought increased ABA biosynthesis and ABA content which confer to *I. walleriana* drought resistance. Drought resistance was also accompanied with increased accumulation of phenolic compounds and carotenoids which in turn increased total antioxidant activity. Activity of antioxidant enzymes SOD, POX and CAT contributed to preventing negative effects of oxidative stress. Considering that *I. walleriana* often suffer from drought stress when grown in containers, this research may be relevant for overcoming this problem in the future. Furthermore, in the context of climate change, global warming and pronounced drought effects from year to year it is very important to pay attention on finding alternative ways to enhance plant productivity under drought stress.
